# Exploiting the potential of commercial digital holographic microscopy by combining it with 3D matrix cell culture assays

**DOI:** 10.1038/s41598-020-71538-1

**Published:** 2020-09-07

**Authors:** Monica Hellesvik, Hanne Øye, Henriette Aksnes

**Affiliations:** 1 Department of Biomedicine, University of Bergen, Bergen, Norway; 2 Department of Biological Sciences, University of Bergen, Bergen, Norway

**Keywords:** Cancer models, Cell culture, Metastasis, Cancer, Cell adhesion, Cell division, Cell growth, Cellular imaging, Cytoskeleton, Cell biology, Cell migration, Cell invasion, Extracellular matrix, Filopodia, Invadopodia, Lamella, Lamellipodia

## Abstract

3D cell culture assays are becoming increasingly popular due to their higher resemblance to tissue environment. These provide an increased complexity compared to the growth on 2D surface and therefore allow studies of advanced cellular properties such as invasion. We report here on the use of 3D Matrigel cell preparations combined with a particular gentle and informative type of live-cell microscopy: quantitative digital holographic microscopy (DHM), here performed by a commercial software-integrated system, currently mostly used for 2D cell culture preparations. By demonstrating this compatibility, we highlight the possible time-efficient quantitative analysis obtained by using a commercial software-integrated DHM system, also for cells in a more advanced 3D culture environment. Further, we demonstrate two very different examples making use of this advantage by performing quantitative DHM analysis of: (1) wound closure cell monolayer Matrigel invasion assay and (2) Matrigel-trapped single and clumps of suspension cells. For both these, we benefited from the autofocus functionality of digital phase holographic imaging to obtain 3D information for cells migrating in a 3D environment. For the latter, we demonstrate that it is possible to quantitatively measure tumourigenic properties like growth of cell clump (or spheroid) over time, as well as single-cell invasion out of cell clump and into the surrounding extracellular matrix. Overall, our findings highlight several possibilities for 3D digital holographic microscopy applications combined with 3D cell preparations, therein studies of drug response or genetic alterations on invasion capacity as well as on tumour growth and metastasis.

## Introduction

It is increasingly recognized that 3D cell culture assays mimic the physiological condition with higher fidelity compared to 2D substrates. In particular in cancer research, various in vitro systems have been developed to enable the study of cancer cell biology in a 3D context. Motility assays, such as in vitro wound healing assays are widely applied to study directional migratory capacity of cancer cells^1^. With the addition of a 3D extracellular matrix, one can more closely mimic the physiological biomechanics of various cellular processes involved in migration that influence on cancer cell invasion mode and the metastatic dissemination of primary cancers^2–4^. In vitro-formed matrix gels is a good cell culture model for cancer cell invasion. It is often used in combination with multicellular spheroids, measuring tumour growth and sprouting^5–10^.

Matrigel is one example of a commonly used reagent that mimics the basement membrane and contains main components of several extracellular matrix (ECM) structural proteins, including collagen IV and laminin^11^. Matrigel forms a gel-like matrix, thereby providing a more complex extracellular environment^12^. In such a three-dimensional landscape, it may be possible for cells to adapt a more in vivo-like migratory mode in which they digest, attach to and navigate through pores of the matrix by proteolysis, integrin expression and intracellular contraction^13,14^, as well as form invading structures^15^. The classical migration assay, in which a scratch wound is made in a confluent cell culture followed by monitoring of the two cell fronts rejoining to close the gap, may be performed in Matrigel as a model for invasion^16–18^ and these types of models are popular in cancer studies, such as for drug discovery^19^.

The most typical microscopy technique used for these types of wound-healing migration or invasion assays, is regular phase contrast and differential interference contrast (DIC) microscopy. An alternative label-free live-cell imaging technique that additionally provides cell 3D information is digital holographic microscopy (DHM), a type of quantitative phase imaging (QPI)^20–22^. The present work focuses on the use of DHM in imaging cell culture populations, which is a recognized accompaniment to higher-magnification super-resolution imaging of subcellular structures^23^. Due to the low light intensity of the laser light used in QPI methods like DHM, live-cell imaging can be performed with frequent image acquisition and is suitable for long-term monitoring of single cells or cell population morphology^24,25^. This microscope technique records a digital hologram of the cell, and this recorded interference pattern is processed computationally to produce a quantitative phase shift image, a holographic image. The digital reconstruction of the hologram is performed numerically in DHM^26^. Therefore, these digital images contain information on biophysical parameters, such as cell thickness, volume and shape, which can be quantified and used to monitor cell morphology^27–29^ in for example phenotype^25^ or drug screens^25,30–33^.

The last decades, QPI has emerged as an important method in biomedical imaging and various QPI techniques have been developed and implemented as tools for biomedical research^22,34,35^. This imaging technology is developing towards a more application-based field^22^ and commercially available QPI systems have recently been made available^36^. In the present study, we used a commercial holographic microscope with integrated software for various applications for quantitative analysis of several cell morphological features in addition to single-cell tracking and wound analysis (see “Methods” section for details).

DHM live-cell imaging is indeed applied in migration studies, both single-cell tracking^37–40^ as well as collective wound migration^36,41–44^. The latter allow for visualization of the migrating cell layer and quantitative motility data based on cell-covered area. A study using digital holographic microscopy provided additional detailed information on morphological features extracted from this type of assay, like cell layer thickness and identification of proliferating single cells from the holographic images^36^. Another study using this system, showed that it was well suited for monitoring and quantitatively assess the motile capacity of cells migrating in a wound-healing assay compared to other established migration assay, like transwell assays^41^. In addition, these authors concluded that this commercial all-in-one DHM system provided advantages such as reproducibility of measurements and compatibility with high throughput applications.

Although DHM is extensively used and optimized for wound-healing migration analysis, the potential of live-cell DHM imaging has, as far as we know, not been extensively explored for invasion studies using dense gel matrices, like thick Matrigel preparations, covering the wound gap. Interference caused by light scattering effects during imaging of such preparations has been indicated as a challenge for DHM imaging^45^, albeit preparations with collagen did prove compatible^45–47^ as well as a Matrigel cluster assay on fixed cells^48^. Cells chemotactically migrating in diluted Matrigel were also trackable using DHM^49,50^. To our knowledge, there are at present limited scientific literature reporting on the performance of the newest commercial DHM imaging technology for wound analysis of cells embedded in thick Matrigel.

The aim of this report was to evaluate the compatibility between a commercial all-in-one DHM imaging system and cell cultures embedded in 3D Matrigel matrix. Hence, we wanted to test whether it would be possible to achieve a double benefit from the strength of monolayer 3D cell preparations and the DHM imaging and its accompanying software application for easy and robust quantifications of cells embedded in and invading into Matrigel.

## Results and discussion

### DHM quantifiable cell morphological and proliferative features in 3D Matrigel matrix

**Figure 1:**
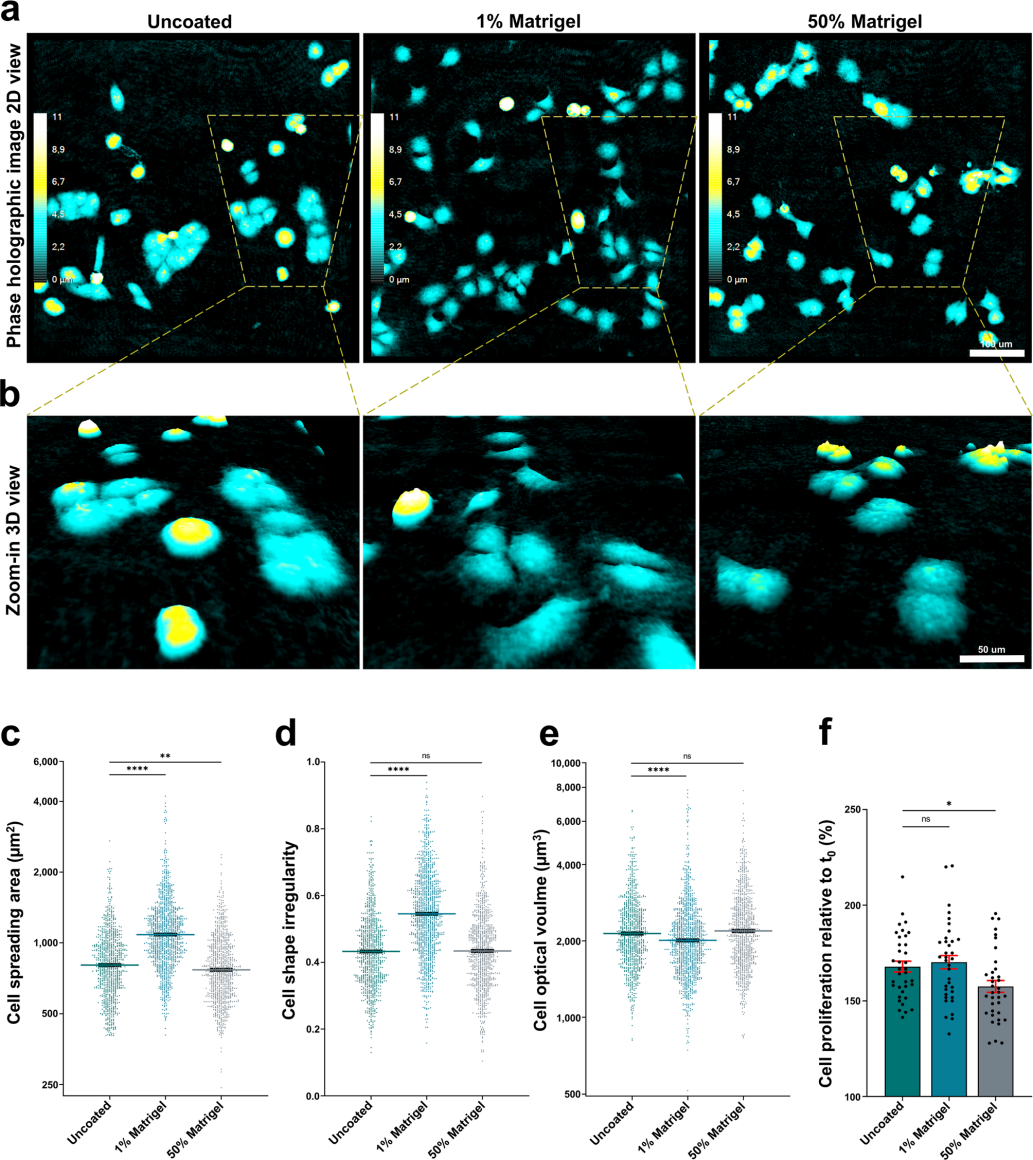
Holographic imaging of Matrigel embedded cells reveal the impact of culture extracellular environment on cell morphology and cell proliferation. (**a**) U2OS cells were prepared for live-cell holographic imaging and seeded on uncoated surface, seeded in 1% Matrigel or embedded in 50% Matrigel followed by image acquisition each 15 min at 20 × for 16 h using the DHM system. The cyan-yellow-white colour bar in the pseudo coloured holographic 2D representation of the 3D images displays a vertical scale (z-plane) correlating the image colouring with optical thickness. (**b**) Zoom-in 3D view revealing morphological differences in U2OS cells as effect of Matrigel in the extracellular environment. (**c**–**e**) Quantification of the morphological features cell spread area, cell shape irregularity and cell optical volume in U2OS cells seeded as in (**a**). Three randomly chosen fields of view from each of three wells per condition were analyzed. Images at timepoint 17.5 h post Matrigel embedding of cells was further processed and analyzed using the integrated software. Data are plotted as lognormal for improved visualization. Original units for the cell morphological parameters are displayed on the y-axis by means of logarithmic scale. Shown is the mean ± SEM of three independent experiments pooled together. Mean of Cell spreading area = 847.7 um^2^(Uncoated); 1,150 um^2^ (1% Matrigel); and mean = 809.3 um^2^(50% Matrigel). Mean of Cell shape irregularity = 0.4326 (Uncoated); 0.5454 (1% Matrigel); and mean = 0.4339 (50% Matrigel). Mean of Cell optical volume = 2,246 um^3^ (Uncoated); 2,130 um^3^ (1% Matrigel); and mean = 2,321 um^3^ (50% Matrigel). n = 897 (Uncoated); n = 1,170 (1% Matrigel); and n = 891 (50% Matrigel). *ns* not significant, *p ≤ 0.05; **p ≤ 0.01; ****p ≤ 0.0001, one-way ANOVA with Dunnett’s multiple comparison test. (**f**) Quantification of cell proliferation comparing relative increase in cell numbers between t_0_ = first image and last image 16 h later acquired as in (**a**). For each condition; single cells in four randomly chosen fields of view from three replicate wells in each experiment were counted from the holographic images using the integrated software. Shown is the mean ± SEM of three independent experiments pooled together. Mean of Cell proliferation = 168% (Uncoated); 170.2% (1% Matrigel); and mean = 157.5% (50% Matrigel). n = 36 (Uncoated); n = 36 (1% Matrigel); and n = 36 (50% Matrigel). *ns* not significant, *p ≤ 0.05, one-way ANOVA with Dunnett’s multiple comparison test.

We initially explored various preparations and challenged the holographic imaging with Matrigel embedded cells in monolayer to evaluate the potential disturbance of thicker culture substrates. Coating of cell culture surfaces and the extracellular environment can have drastic effects on cell behavior and morphology, so we were interested to determine whether the commercial DHM system could decipher these. U2OS cells were prepared for live-cell holographic imaging and seeded under three different conditions: (1) on non-coated surface; (2) in 1% Matrigel and (3) embedded in 50% Matrigel (Fig. 1). Note that contrary to the 50% Matrigel, 1% Matrigel does not form a 3D meshwork for cells to navigate in, but rather coats the dish similar to laminin.

Importantly, holographic imaging was not notably affected in its ability to detect cells and cellular shapes by the presence of a thicker Matrigel preparation (50%) (Fig. 1a, b, Supplementary Fig. S1, Supplementary Movie S3). We found it surprising how well the holographic imaging could handle increasing thickness of gel preparations, with possibly suboptimal refractive indices and potential for reading it as noise^24^. This allowed visual evaluation of the effects of the surface/extracellular environment in addition to a quantitative analysis (Fig. 1c–e). U2OS cells responded most dramatically to the 1% Matrigel condition. Here, cells were clearly more spread on the surface (Fig. 1a–c, Supplementary Movie S2) compared to cells in uncoated wells (Supplementary Movie S1), thus adapting a flatter morphology with bow-shapes reflecting actively migrating cells, as also indicated by an increased tendency to separate from neighbor cells as well as increased cell shape irregularity (Fig. 1d) and reduced optical volume (Fig. 1e). As expected, cells in 50% Matrigel spread out less than the control situation with 1% Matrigel, but rather displayed a higher optical thickness (Fig. 1a–c and Supplementary Movie S3). Examples of cells with pointy protrusions were also observed in the Matrigel samples, possibly reflecting a 3D-adapted mesenchymal migratory mode. This indicated the advantage of obtaining 3D image information from cells in 3D cultures. However, detailed analysis of fine protrusions is not possible with the 20 × objective used here. Protrusion-analysis was indeed previously performed on Matrigel/collagen embedded cells, there imaged with 40 × using a QPI-technique called CCHM (coherens-controlled holographic microscopy)^47^.

With its ability to detect single cells, DHM is well qualified to measure cell proliferation^41,51^. We additionally analyzed cell proliferation using the same images and found that there was a visually lower proliferation rate for cells in 50% MG compared to uncoated surface and 1% Matrigel (Fig. 1a, b) and Supplementary Movie S1 (uncoated) vs Supplementary Movie S3 (50% MG). The corresponding quantitative difference is shown in Fig. 1f. Decreased cell proliferation in ECM preparations was also measured previously for the same cell line.

### DHM quantified single-cell migratory tracking in 3D Matrigel matrix

**Figure 2:**
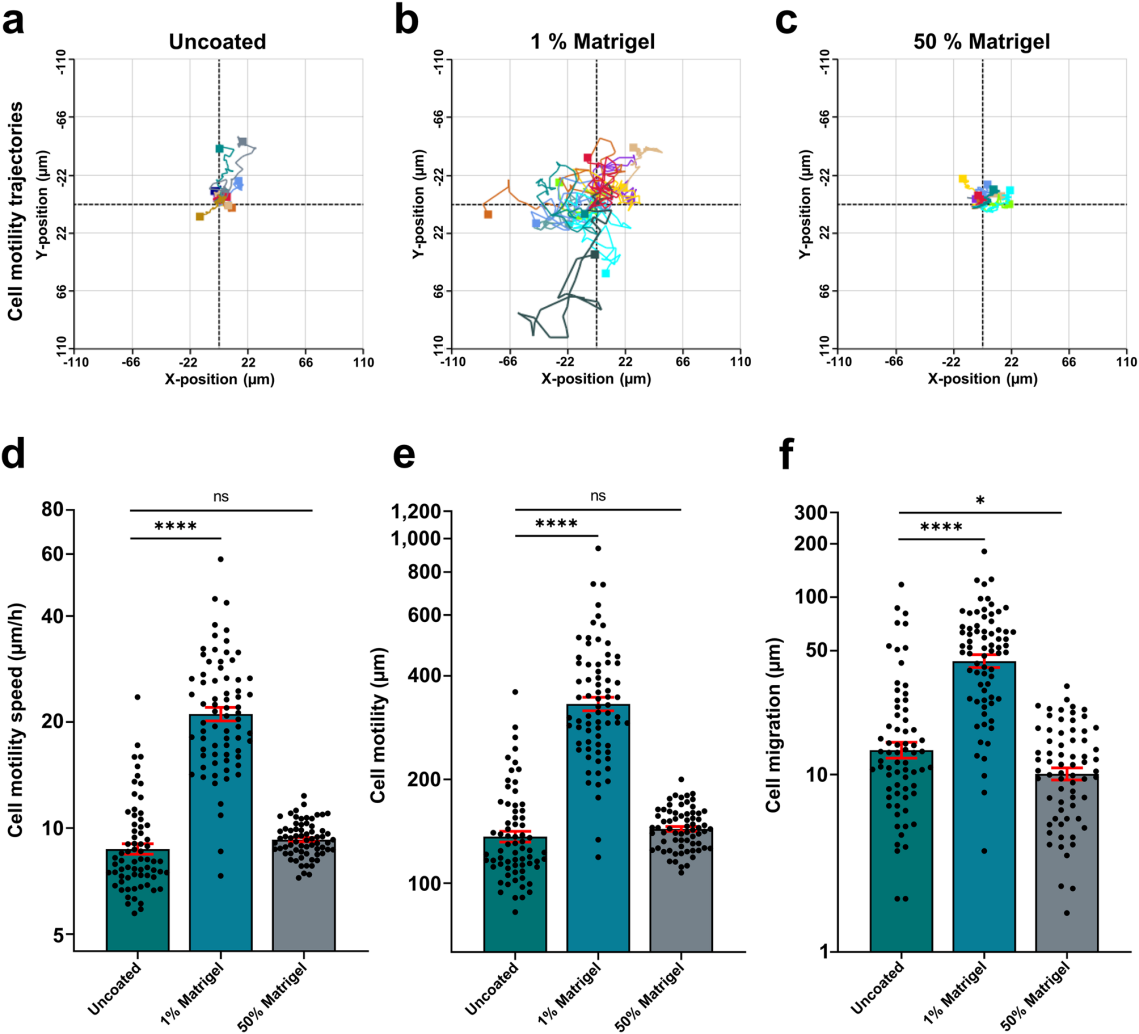
Holographic imaging of cells reveal the impact of culture extracellular environment on cell migration and cell motility. U2OS single cell tracking analysis performed with the integrated software from the phase shift holographic images acquired as described in Fig. 1. Cells were prepared for live-cell holographic imaging and seeded on uncoated surface, seeded in 1% Matrigel or embedded in 50% Matrigel followed by image acquisition every 15 min at 20 × for in total 16 h using the DHM system. (**a**–**c**) Single cell 2D movement trajectories from 10 to 13 cells per field of view were displayed as a rose plot. Each plot represents migration path over 16 h. (**d**–**f**) Cell motility speed, cell motility and cell migration were quantified from single cell tracking data. Motility distance was defined as the accumulated movement of the cell over time from the starting point to the end point of the cell path. Migration was defined as the shortest distance from the starting point and the end point of the cell path. Each data point represents the quantitative data from tracking a single cell throughout 16 h of imaging. Between 10 and 13 cells from one randomly chosen field of view from two wells per condition were analyzed using the integrated software. Data are plotted as lognormal for improved visualization. Original units for cell movement parameters are displayed on the y-axis by means of logarithmic scale. Shown is the mean ± SEM of three independent experiments pooled together. Mean of Cell motility speed = 9.115 µm/h (Uncoated); 22.57 µm/h (1% Matrigel); and mean = 9.328 µm/h (50% Matrigel). Mean of Cell motility = 143.3 µm (Uncoated); 356.8 µm (1% Matrigel); and mean = 145 µm (50% Matrigel). Mean of Cell migration = 20.3 µm (Uncoated); 54.40 µm (1% Matrigel); and mean = 12.13 µm (50% Matrigel). n = 69 (Uncoated); n = 72 (1% Matrigel); and n = 68 (50% Matrigel). *ns* not significant, *p ≤ 0.05; ****p ≤ 0.0001, one-way ANOVA with Dunnett’s multiple comparison test.

We further found that the useful application in our commercial DHM system performing single-cell tracking for migration trajectories was similarly compatible with cells embedded in thick Matrigel (Fig. 2). As shown in the rose plots of single cell migratory paths (Fig. 2a–c) as well as the cell motility speed (Fig. 2d), cells were most motile in the 1% Matrigel condition. Although the cellular motility speed was not different between the uncoated and the 50% Matrigel condition, zoomed-in rose plots reflected a rather distinguishable movement type of cells in the thick Matrigel preparation (Supplementary Fig. S2). The cells in 50% Matrigel only had very subtle movements, producing compact rose plots. We further took a look at the quantified values for motility (total length of cell path) and migration (shortest distance from the starting point to the end point of the cell path) and found that the visual difference between the 50% Matrigel and uncoated conditions displayed in the rose plots was reflected in these numbers. Cells in 50% Matrigel had a significantly shorter migration length, but not motility. Hence, this analysis again showed that ECM components and the % of Matrigel caused a highly altered cell phenotype, as also visible in the Supplementary Movies 1–3.

### Wound-healing invasion in monolayer 3D preparation analyzed with commercial DHM system

**Figure 3:**
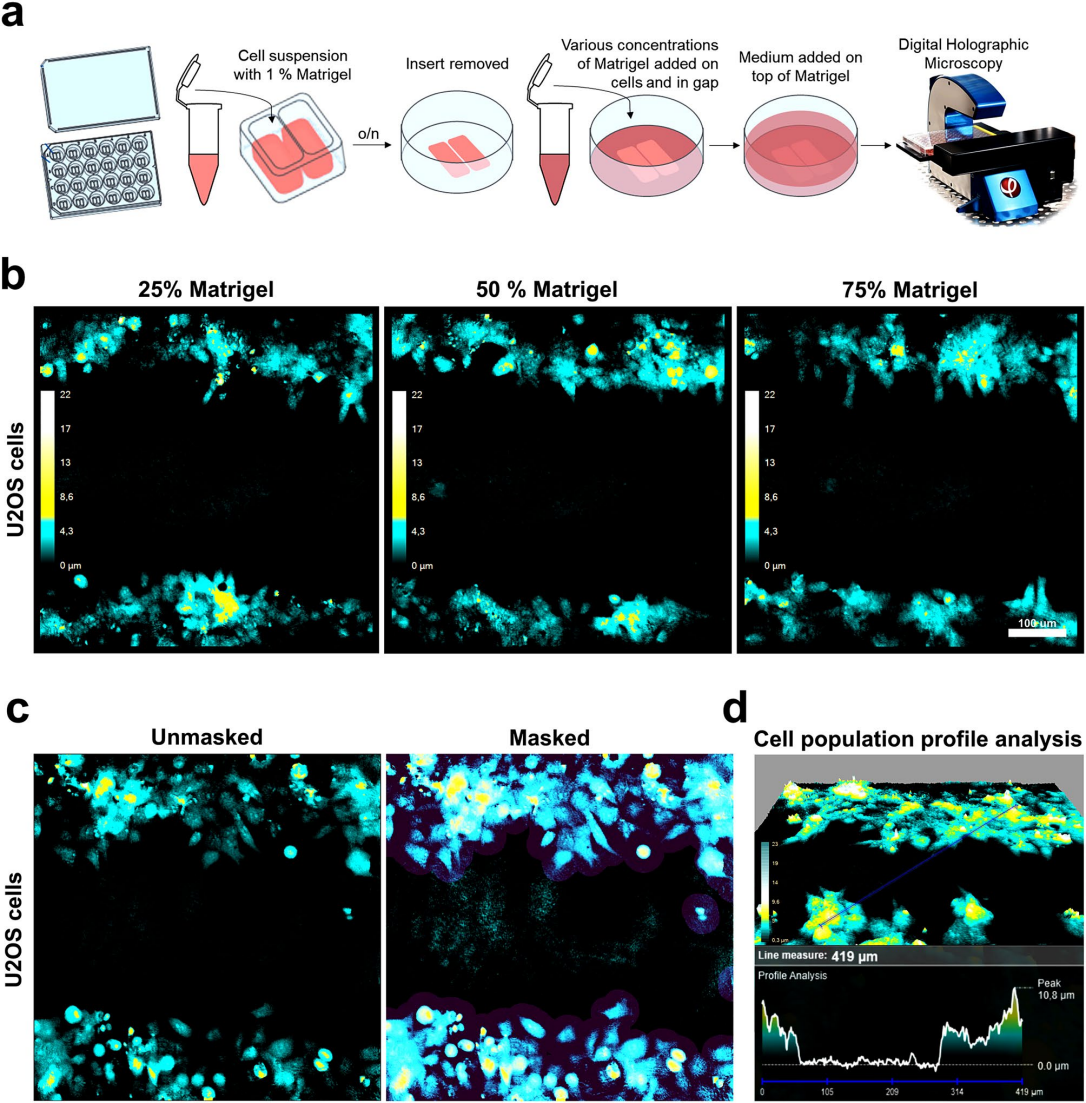
Matrigel wound creation and analysis. (**a**) Schematic overview of creation of Matrigel wound for invasion assay. (**b**) Holographic imaging of cells and gap embedded in various concentrations of 3D Matrigel matrix, 25%, 50% and 75%. U2OS cells invading into the 3D matrix were monitored for 30 h. The cyan-yellow-white colour bar in the pseudo coloured holographic 2D representation of 3D images displays a vertical scale (z-plane) correlating the image colouring with optical thickness. (**c**) Example of holographic image from U2OS cells invading Matrigel (50%), before (left) and after (right) application of the image mask used for area measurement of cell coverage and quantification of gap closure over time. (**d**) Visualization of the topographic profile of the invasive population-landscape of U2OS cells correlated to changes in optical thickness.

Many commercial systems have built-in applications for various analyses including wound healing^40^. Here, we took advantage of an integrated wound analysis tool that facilitates efficient quantifications in wound healing assays^41^, such as used to demonstrate effects of substances and hypoxia on cell migration^52^. Based on the above results, we next wanted to check whether it would be possible to use this migration analysis tool on our commercial DHM system to perform a cell culture wound assay to measure invasion capacity in 3D Matrigel (Fig. 3). We prepared 3D Matrigel wounds for invasion analysis, as depicted in Fig. 3a. First, we performed an initial test to challenge the imaging capacity of the system with increasing Matrigel concentrations. Here, we covered the cell gap with three different concentrations of a thick layer of Matrigel (25%, 50% and 75%). Interestingly, we found that all Matrigel concentrations tested were indeed compatible and did not interfere with the image acquisition on this holographic imaging device (Fig. 3b). Moreover, the Matrigel did not interfere with the function of the built-in analysis tools to apply mask on the image for cell area detection (Fig. 3c) or detection of the topographic profile (Fig. 3d).

**Figure 4:**
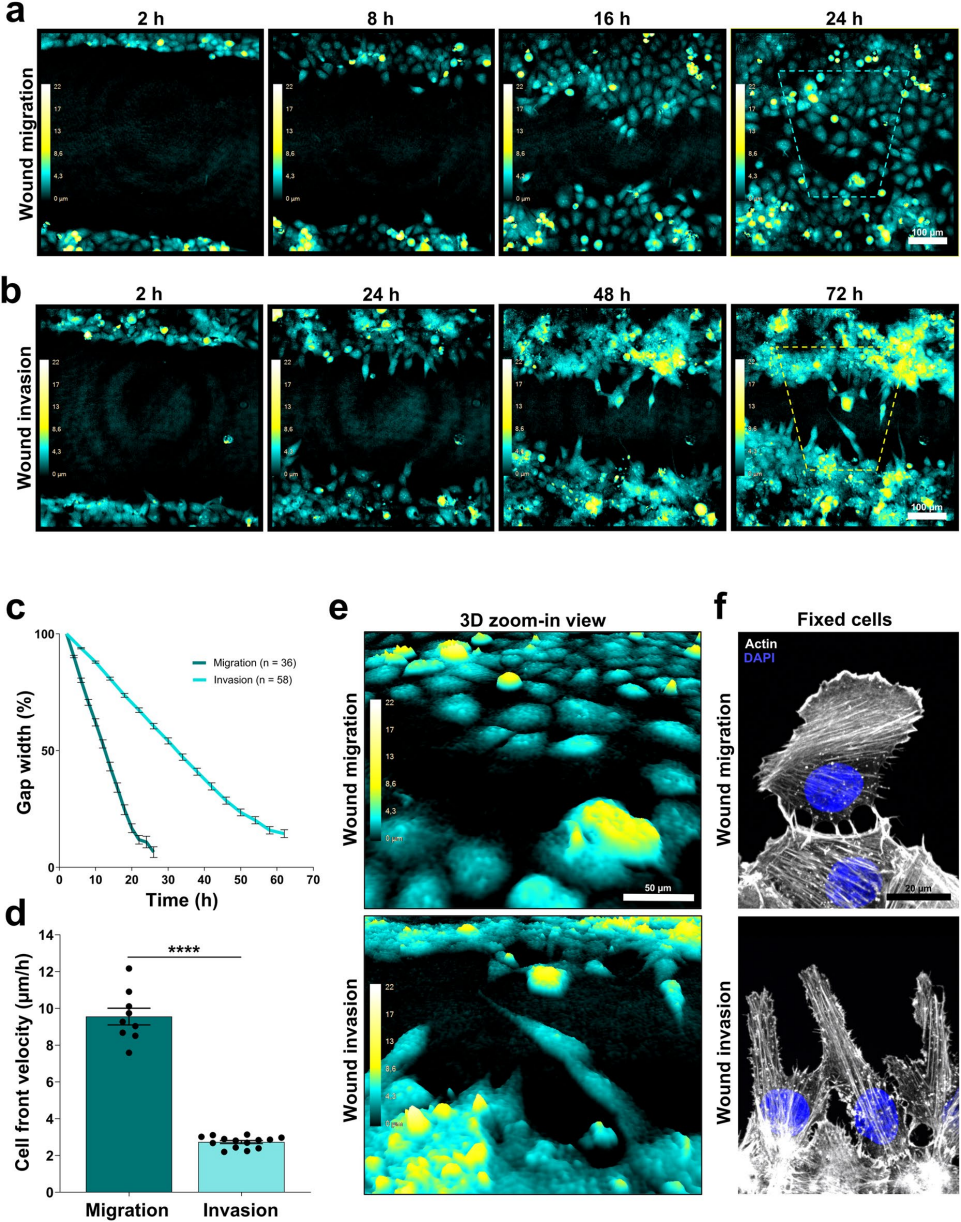
U2OS cells in Matrigel wound analyzed for invasive or migratory capacity with digital holographic imaging. (**a**–**c**) U2OS cells were prepared for wound healing assays. For wound migration; three fields of view in each well in three replicate wells per experiment were imaged at 20 × every 15 min for 26 h using the DHM system. Wound gap and cells in the wound invasion assay were embedded in 50% Matrigel and 5 fields of view from each well were acquired at 20 × every 15 min for 72 h. Images were further processed and analyzed using the built-in software. (**a**) Representative images from wound migration showing the degree of gap closure at 2, 8, 16 and 24 h after gap creation. The cyan-yellow-white colour bar in the pseudo coloured holographic 2D representation of 3D images displays a vertical scale (z-plane) correlating the image colouring with optical cell thickness. (**b**) Representative images from wound invasion showing the degree of gap closure at 2, 24, 36 and 48 h. Colour bar as in (**a**). (**c**) Quantification of gap width closure relative to gap width where t = 2 was set to 100%. Data was collected from images acquired every second hour (wound healing migration) or fourth hour (wound healing migration) throughout the time lapse. For wound healing migration; shown is the mean ± SEM of data from three fields of view per well in three replicate wells per experiment and three independent experiments pooled together, with n = 36 wound fields in total. For wound healing invasion; shown is the mean ± SEM of data from three to five fields of view per well in four or five replicate wells per experiment and three independent experiments pooled together, with n = 58 wound fields in total. (**d**) Cell front velocity was calculated from the linear phase of the slopes from (**c**). Wound healing migration; shown are the mean ± SEM of data from the average of three fields of view per well in three replicate wells per experiment, and three independent experiments pooled together, with n = 9 in total. Wound healing invasion; shown are the mean ± SEM of data from the average of three to five fields of view per well in four or five replicate wells per experiment, and three independent experiments pooled together, with n = 14 in total. (**e**) Zoomed-in 3D visualization of leading front morphology of U2OS cells indicated with dotted line frames in (**a**) and (**b**). (**f**) Zoom-in view of leading front morphology of U2OS cells fixed 12 h post gap creation. F-actin were labelled with fluorescent phalloidin and nuclei stained with DAPI. Images were acquired with spinning disk confocal at 100×.

Following our Matrigel %-titration setup for compatibility with imaging and analysis on our commercial all-in-one DHM system, we attempted a wound healing invasion assay on U2OS cells in 50% Matrigel, paralleled by a classical wound healing migration assay with a non-ECM covered gap (Fig. 4). We found that U2OS cells invaded the Matrigel and was able to close the wound gap within the 72 h of imaging (Fig. 4a, c, Supplementary Movies S6–S7), corresponding to an invasion speed of 2.8 μm/h (Fig. 4d). This was comparable to previous data of U2OS cells in similar ECM-like invasion assays^53^. Migration in an untreated cell gap was significantly faster than the invasion in 50% Matrigel; 9.6 μm/h. We also noticed differences in migration mode (Supplementary Movies S4–S7) and cell morphologies between migrating and invading cells. This likely reflects an invasive migration mode and possibly a mesenchymal migratory mode, typically characterized by cells having an elongated spindle-shaped cell morphology and being dependent on ECM proteolysis by MMPs to create small micro-tracks^54^. In the videos (Supplementary Movies S6–S7) we observed a higher degree of a form of collective migration in the 50% Matrigel matrix in which leader cells are followed by neighbor cells seemingly squeezing through the trail, whereas in non-ECM covered wounds, the migration seems more “loose” in the front, i.e. with less contact between leader and follower cells as well as typically with an apparent broad bow-shaped lamellipodia (Fig. 4a, e, f and accompanying Supplementary Movies S6–S7). The advantage of the autofocus function of DHM imaging proved useful in obtaining 3D information for cells migrating in a 3D environment. Overall we conclude that it is indeed possible to expand the use of the wound-healing application in this DHM system from migration analysis to invasion analysis since it proved compatible with wounds covered with a layer of thick (50%) Matrigel.

### DHM analysis of suspension cells embedded in Matrigel can quantify metastatic capacity

**Figure 5:**
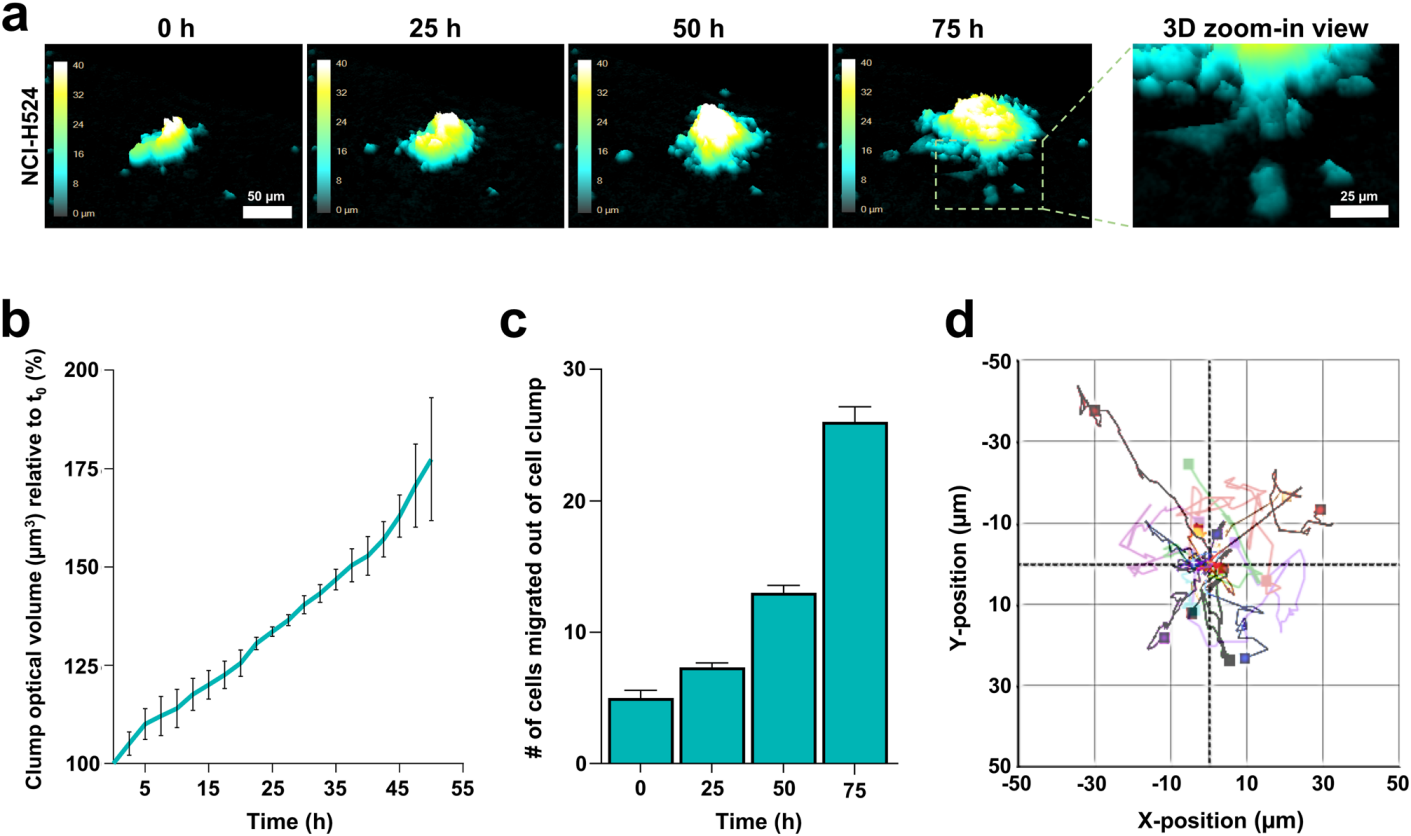
Holographic live-cell imaging and analysis of suspension cells embedded in Matrigel. (**a**) Representative images of lung cancer cell line NCI-H524 embedded in 50% Matrigel acquired using live-cell digital holographic microscopy (DHM) imaging at 20 × for 75 h. The cyan-yellow-white colour bar indicates height ranging 0–45 µm in the z-plane in this 2D representation of the 3D images. Accompanying 3D zoom-in view shows single cells emerging out of their respective cell clumps. (**b**) Cell clump growth over time. Analysis of DHM time-lapse imaging as shown in (**a**) in which optical volume was measured every 15 min for a total of 50 h. Shown is the mean ± SEM from two independent experiments pooled together (n = 3 cell clumps). (**c**) Single-cell migration out of cell clump shown as number of cells counted at given time points 0, 25, 50 and 75 h, t_0_ = 24 h post-seeding. Shown is the mean and error bars representing SEM (only upper bar indicated) from two independent experiments pooled together, where n = three cell clumps monitored for single cell migration. (**d**) Rose plot showing single cell 2D movement trajectories from two independent experiments. 6 to 11 single cells from each of two different fields/cell clumps, were randomly chosen and subsequently tracked for 12 h.

We further took advantage of the compatibility of Matrigel and our commercial DHM system, to explore options for monitoring suspension cells in this system. Floating freely in multiple levels in the media, suspension cells represent a challenge for microscopy. Also, live-cell imaging for quantitative analyses requires multiple fields and wells to be monitored. This is achieved by a motorized automated stage. Although for fixed applications, suspension cells may be attached to coverslips by cytocentrifugation such as cytospin technology^55^, a live-cell imaging setup is normally not compatible with suspension cells. DHM has previously been performed on live suspension cells immobilized by antibody capture^56^. Here, we tested the option to use thin 3D gels to immobilize suspension cells for multifield monitoring. Such ECM embedding of suspension cells has been described earlier to work well with lung cancer suspension cells^57^. Here, we used the lung cancer cell line NCI-H524, normally grown in suspension in liquid RPMI 1640. The suspension cells were embedded in 3D Matrigel as described in the “Methods” section. This approach allowed us to analyse several properties of the suspension cells with the commercial DHM device (Fig. 5). The NCI-H524 cells grow both as single cells and as clumps while in suspension culture. By monitoring Matrigel-immobilized cell clumps, we could measure clump size or 3D optical volume of the clump over time (Fig. 5a, b). Interestingly, we found that once exposed to the Matrigel, the NCI-H524 cells started to migrate away from the clump by invading the surrounding Matrigel matrix (Fig. 5a). This allowed analysis of single-cell migration and an estimate of the migration speed (Fig. 5c, d). These types of tumour behavioural analyses on growth, as performed on these spontaneously formed cell clumps, could probably be applied for spheroid assays as well^58^, and additionally open the possibility for a quantitative measure of metastasis from spheroids. Note that the commercial system used here was developed for adhesive cells in monolayers. Consequently there is a limitation for the size of the spheroid as it, along with the single cells leaving it, would need to be within the size of the image field (500 × 500 µm), and in the z-plane the limit is around 50 µm for the system used here. DHM systems designed specifically to analyze thick specimens like spheroids do exist.

## Conclusion and future perspectives

We here demonstrated the compatibility of Matrigel preparations with a commercial software-integrated system for digital holographic microscopy and quantitative analysis. Neither image quality nor quantitative analysis was notably affected, even by thick Matrigel preparations (50–75%). We took advantage of this and challenged the integrated wound-healing analysis tool that is normally used for migration studies, with analysis of invasion capacity in Matrigel-covered cell gap. By further taking advantage of the compatibility of this DHM system with Matrigel, we performed analyses of matrix-immobilized suspension cells and were able to measure two interesting features connected to tumour biology: (1) growth of cell clump over time, and (2) single-cell invasion out of cell clump and into the surrounding Matrigel matrix.

Some limitations of the DHM system should be noted. As a cell population-based instrument with a 20 × objective, this DHM system has limitations regarding detection and quantification of fine protrusions as possible with other systems^47^. Nor is it possible to distinguish intracellular organelles (besides the nucleus to some degree), something which has been done with other QPI systems^59^. Additionally, the commercial system used here is suitable for high-throughput data analyses and is developed for adhesive cells in monolayers. We have shown here that it is possible to challenge this system past adhesive monolayers with a good performance analyzing cells in 3D Matrigel in all applications tested. Nevertheless, there is a limit as cells or cell clumps must not exceed a certain size range. It should also be noted that the experiments performed here were all on cell monolayers embedded in 3D matrix. A true tracking of cells in a full 3D environment in which cell migration can also be followed in z- in addition to x/y-plane^45,46,60^, is not possible with the DHM system reported on here. The strong benefits of the commercial DHM system used here, is the all-in-one integration with a user-friendly accompanying software with a multitude of analysis options and quantifiable cell parameters.

3D cell culture advancements are becoming increasingly popular in all aspects of cell biological research. Our findings expand the use of digital holographic microscopy (DHM) designed for monolayer adhesive cells to reveal novel characteristics of cancer cell invasion in 3D and highlight its potential to contribute to our understanding of invasive mechanisms through for example large-scale studies of drug response on invasion capacity as well as on tumour growth and metastasis.

## Methods

### General cell culture

U2OS cells were cultured in DMEM supplemented with 10% FBS and 1% Penicillin/Streptomycin and kept at 37 °C, 5% CO_2_ with humidified atmosphere and passaged approximately every 2–3 days. Cells were mycoplasma tested and fingerprinted.

The suspension cell line, NCI-H524 (ATCC CRL-5831) was cultured in Nunc Non-treated Flasks (T 25 cm^2^/T 75 cm^2^) using RPMI 1640 supplemented with 10% FBS and 1% Penicillin/Streptomycin (complete media) and kept at 37 °C, 5% CO_2_ with humidified atmosphere.

### Quantitative phase imaging with digital holographic microscopy using HoloMonitor M4

The HoloMonitor M4 is a small time-lapse cytometer used for label-free live-cell imaging and quantitative analysis of monolayers of adhesive cells. The HoloMonitor M4 unit is equipped with a motorized xyz-stage, an Olympus PLN 20 × microscope objective, a light source in an external low-power laser unit (635 nm wavelength, 0.2 mW/cm^2^) and a 1.3 MP CMOS global shutter USB 2.0 camera^61^. The technique employed, digital holographic microscopy, is based on measurements of phase shifts detected when a laser beam pass through living cells^32^. The incoming laser is split into two beams, the sample beam and the reference beam. When the sample beam illuminates the cell, the light gets distorted when it passes through the cell, creating waves of light or phase shifts. When these waves are rejoined with the reference beam, an interference pattern, a hologram, is detected by the digital image sensor. Based on the hologram, a cell image or holographic image is reconstructed using a computer algorithm^51^. This quantitative phase imaging method is used for analysis and quantification of various cellular features, like cell movement and parameters for cell morphology^24^.

### Analyzing cell morphology, proliferation, motility and migration using live-cell holographic imaging and Matrigel

U2OS cells (40,000 cells/ml) were seeded into an ibidi 24-well ibiTreat plate (500 µl/well) with uncoated wells. I addition, cells mixed with 1% Matrigel (stock solution 10.8 mg/ml, diluted in cell culture medium) were seeded into uncoated wells. Cells were allowed to attach for 20 min in RT following 40 min incubation in 37 °C, 5% CO_2_ in a humidified incubator. After attachment, cells were carefully washed once with 1 ml culture medium. 1 ml culture medium was added again and the 24 well plate was equilibrated on ice for 5 min. Medium was carefully removed and four different extracellular conditions were generated as follows: (1) 50% Matrigel layer (300 µl/well) was added on top, embedding the cells. (2) 2.5 ml culture medium mixed with 1% Matrigel were added to uncoated wells or (3) 2.5 ml culture medium was added on top of cells in uncoated wells. After 30 min of polymerization in 37 °C allowing formation of a thick gel in wells containing 50% Matrigel, it was gently overlaid with cell culture medium (2.2 ml/well). The 24-well plate was then calibrated with HoloLids for ~ 15 min at 37 °C, 5% CO_2_. Live-cell holographic imaging was set up according to manufacturer’s manual as described previously^37^. Three fields of view were monitored at 20 × magnification per well. Images were acquired every 15 min, for 16 h in total.

Image processing and analysis were performed with Hstudio software from the holographic image acquired ~ 17.5 h post Matrigel polymerization. The software identifies cells using a watershed based segmentation algorithm^61^. First, automatically initial preset values for threshold and regional maxima was suggested by the software. Further optimization of segmentation of cell spread morphology and single cell identification was achieved by applying Auto-Otsu background threshold, pre-smoothing “off” and adjustment of object size threshold in the Hstudio software. Lastly, manual adjustments were used to optimize the automatic identification of cell perimeter, e.g. deleting non-cellular objects background or manual acquisition of cell spread morphology where automatic identification was regarded as non-optimal. In addition, to deselect cell debris, cells rounding up before division and newly divided cells, a minimum threshold level was set to 400 µm^2^. After the identification step, cells situated at the image edge were automatically excluded from the analysis, and the remaining identified cells could be subjected to quantitative analysis of morphological parameters such as cell spread area, optical volume and cell shape irregularity, performed by the integrated software. Three fields of view per well in three replicate wells per condition were analyzed, and in total three individual experiments were performed.

#### Parameters used for quantification of cell morphology

Several morphological parameters of the cell may be obtained from the reconstructed hologram: “Area” is calculated from the total number of pixels used to image the surface area covered by the cell^32,61^. The thickness of the cell, “Optical thickness”, in a given pixel is obtained from the phase shift, the wavelength of the laser light and the refractive index of the cell and surrounding medium^62^ The “Optical volume” is the estimated volume of a cell calculated from the phase shift and is independent of its shape^40^. The volume can be calculated from the area and the thickness of the cell^32^. “Irregularity” is a measure of how much the circumference of the cell deviates from the circumference of a perfect circle. The value “0” characterizes a circular cell and when the irregular outline of the cell increases, the irregularity value becomes higher, approaching “1”^61^.

#### Quantification of cell proliferation

To assess the difference in U2OS cell proliferation in various extracellular environments, single cells were counted from the holographic images acquired as described earlier. A relative comparison was made from the increase in cell numbers between t_0_ = first image and last image 16 h later. The “Identify cells” module in the Hstudio software were used for single cell identification and cell counting. Single-cell identification was performed automatically using Auto-Minimum Error background threshold and pre-smoothing “on”. In a few cases, when cell identification was regarded as non-optimal, cell count was adjusted manually. Cells situated at the image edge were automatically excluded from the analysis. For each condition, uncoated, 1% Matrigel and 50% Matrigel, single cells from four randomly chosen fields of view per well from three replicate wells in each experiment were counted. Quantitative data from three individual experiments were analyzed.

#### Single cell tracking and analysis of cell motility and cell migration

Image processing and analyzes of cell motility parameters were performed with Hstudio software from the same images acquired for cell morphology parameter analysis. Single-cell identification was performed automatically using Auto-Minimum Error background threshold and pre-smoothing “on”. Further optimization of cell identification was achieved using manual adjustment of Auto-Minimum Error threshold and object size threshold level. These adjustments were applied for all images throughout a time-series of about 60 images with 15 min interval between each image, covering 16 h imaging in total. When automatic thresholding was regarded as non-optimal, manual adjustments were applied on single cells in individual images in the time lapse series. Images with insufficient quality for identification and analysis were manually deleted. For each field of view, between 10 and 13 single cells were identified and cell movement were tracked every 15 min using Hstudio “Track cells” module throughout a times series of ~ 60 frames, covering 16 h imaging in total. Single cell movement trajectories were presented as rose-plots. Quantitative cell motility parameters as migration, motility and motility speed was collected from the cell tracking data set. Motility was calculated by the software as the accumulated movement from the starting point to the end point of the cell path during the investigated time period. Migration was calculated as the shortest distance from the starting point at t_0_ and the end point of the cell path. One field of view per well in two replicate wells per condition were analyzed from in total three individual experiments.

### Wound healing Matrigel invasion assay

U2OS cells (300,000 cells/ml) were mixed with 1% Matrigel (stock solution 10.5 mg/ml, diluted with cell culture medium), seeded in ibidi two-chambered silicone inlet (90 µl/chamber) on a 24 wells ibiTreat plate and incubated o/n. The following day, inserts were removed to create a wound or cell gap and then cell patches were washed 2 × with 1 ml culture medium. 1 ml culture medium was added again, and the 24-well plate was equilibrated on ice for 5 min before 50% Matrigel layer (300 µl/well) was added on top, covering cells and the cell gap (Fig. 3a). After 30 min of polymerization in 37 °C allowing formation of a thick gel, it was gently overlaid with cell culture medium (2.2 ml/well). The 24-well plate was then calibrated with HoloLids for ~ 1 h at 37 °C and invading cells were imaged with the HoloMonitor M4. 6 replicate wells were used, and 5 fields were monitored per well. Time-lapse imaging was set up for acquisition every 15 min, for 72 h in total. Image analysis was performed with Hstudio software using the “Wound healing” module. Here, as seen in Fig. 3c, this integrated wound healing tool automatically detects the cell covered area by applying a mask based on the thresholding method “Adaptive tophat”. If needed during the analysis, this mask was also manually adjusted to optimize identification of the progressing cell regions. The area of the gap was calculated for every 4th hour. Some fields were excluded due to technical issues such as cell wall failure, cell wall obstruction and non-optimal focus for segmentation of cell covered area; leaving 3–5 fields of view per well, 4 or 5 replicate wells useable for the analysis per experiment. In total three individual experiments were performed and analyzed.

### Wound healing migration assay

U2OS cells (300,000 cells/ml) were mixed with 1% Matrigel (stock solution 10.5 mg/ml, diluted with cell culture medium) and seeded in ibidi two-chambered silicone inlet (90 µl/chamber) on a 24 well ibiTreat plate and incubated o/n. The following day, inserts were removed to create a wound or cell gap and then cell patches were washed 3 × with 1 ml culture medium, before adding 2.5 ml culture medium containing 0.2% Matrigel, per well. The 24-well plate was then calibrated with HoloLids for 30–45 min at 37 °C before migrating cells were imaged with the HoloMonitor M4. Three fields of view per well in three replicate wells were monitored and time-lapse imaging was set up for acquisition every 15 min for 26 h in total. Images were analyzed as described in the wound healing Matrigel invasion assay, accept gap closure was calculated for every 2nd hour. In total three fields of view per well from three replicate well for each experiment were analyzed.

### Phalloidin based staining of cells in wound gaps

To assess the state of single cell morphology during wound healing invasion and migration, cells were seeded and wound created as described for the wound healing migration and invasion assay (phenol free Matrigel), but adjusted to an ibidi 8-well chambered glass bottom coverslip. 12 h post gap creation, cells were fixed, permeabilized, washed and stained as follows: Cells were fixed in 3% PFA phosphate buffer for 25 min (migration) or 35 min (invasion) at RT, keeping the ibidi 8-well chambered glass bottom coverslip on a pre-heated metal block (37 °C). After fixation, cells were washed three times with PBS and permeabilized using 0.1% Triton X-100 for 10 min, followed by three washes with PBS. Cells were stained with fluorophore-conjugated phalloidin (1:50, diluted in PBS) directly into the wells and protected from light during 45 min of incubation. After three washes with PBS, DAPI (300 nM, diluted in PBS) were added for 5 min, followed by 3 × PBS wash.

### Spinning disc confocal microscopy

Zoom-in representation of single cell morphology were obtained from wound healing assays using phalloidin-488 for detection of F-actin cytoskeleton and DAPI for nucleus. Images were acquired as 2 × 2 multifield z-stacks at 100×, step size 0.3 µm (migration) or step size 0.1 µm (invasion) using Andor Dragonfly 500 for high-speed confocal imaging. The spinning disk confocal system was mounted on a Nikon Eclipse Ti2 microscope with a CFI SR HP Apo TIRF 100 × 1.49 NA oil objective, iXon3 EMCCD camera and appropriate filter combinations. The system was coupled to a multi-line laser source, the Andor Integrated Laser Engine, in combination with the Andor Borealis enhanced CSU Beam Conditioning Unit. Images were acquired using Fusion software, multifield images was generated by Andor Fusion Stitcher and images were further processed in Imaris software.

### Suspension cell assay using Matrigel and holographic imaging

NCI-H524 suspension cells previously cultured in liquid medium were seeded in 50% Matrigel (stock solution 10.8 mg/mL). Here, a thin gel was prepared (50 µL/cm^2^) by diluting with cell suspension and placed in each 35-mm ibidi µ-cell culture dish. After 30 min of gel polymerization at 37 °C, 5% CO_2_ in a humidified incubator, cells in gel was gently overlaid with 3 mL cell culture medium and calibrated with sterilized HoloLids for 30 min at 37°, 5% CO_2_. Live-cell holographic imaging was set up according to manufacturer’s manual, and a minimum of three fields of view each showing a clump of NCI-H524 cells were monitored at 20 × magnification selected from two independent experiments. Images were acquired every 15 min, processed and analyzed using Hstudio software. Cell clumps were identified using Auto-Otsu thresholding, pre-smoothing “on” and single cells were identified using Auto—Minimum Error thresholding, pre-smoothing on. Automatic cell identification was further improved by manual adjustments of threshold levels and adjusting object size threshold. At last, manual changes were used for additional optimization of the identification of cell perimeter, e.g. deleting non-cellular objects background. After the identification step, segmented single cells or clump could be subjected to quantitative analysis. Two individual experiments were performed. From these experiments the following was quantified: In total three cell clumps were analyzed from three different fields of view from in total two replicate wells. In addition, four to 26 single cells migrating out of the three clumps were manually counted at time points 0, 25, 50 and 75 h after imaging was started. From two of the clumps; six to 11 single cells per field of view were tracked over 12 h from in total two fields of views.

### Generation of time-lapse movies from holographic images

Representative time-lapse movies accompanying Figs. 1 and 4 is found in Supplementary information (7 movies altogether). Time-lapse movies S1–3 of U2OS cells on uncoated surface, seeded in 1% Matrigel or embedded in 50% Matrigel were imaged at 20 × using the HoloMonitor M4 system. Images were acquired every 15 min for 16 h and Hstudio software with “Export Movie” module was used to generate ~ 30 MB movies, using 6 fps. Time-lapse movies S4–5 of U2OS cells performing wound healing migration were imaged every 15 min at 20 × for 26 h and yielded a ~ 43 MB movie (2D) and a ~ 36 MB movie (3D) made by 12 fps. Time-lapse movies S6–7 of U2OS cells performing wound healing invasion into 3D Matrigel imaged every 15 min at 20 × for 72 h and yielded a ~ 120 MB movie (2D) and a ~ 100 MB movie (3D) made by 12 fps.

### Statistical analysis

GraphPad Prism v8 was used to assess the statistical significance of differences evaluated by an unpaired two-tailed t-test with Welch correction or one-way ANOVA with Dunnett’s correction for multiple comparisons. Data normality was assessed using the D’Agostino-Pearson test. If the data sets were shown to more likely have been sampled from a lognormal distribution, the data were transformed by taking the logarithm of each value to approach a Gaussian distribution.Values presented in the figures represent mean ± SEM. Results were considered to be statistically significant if *p* ≤ 0.05.

## Supplementary information

Supplementary Legends.

Supplementary Figure S1.

Supplementary Figure S2.

Supplementary Video S1.

Supplementary Video S2.

Supplementary Video S3.

Supplementary Video S4.

Supplementary Video S5.

Supplementary Video S6.

Supplementary Video S7.

## References

[1] Rodriguez LG, Wu X, Guan JL (2005). Wound-healing assay. Methods Mol. Biol..

[2] Clark AG, Vignjevic DM (2015). Modes of cancer cell invasion and the role of the microenvironment. Curr. Opin. Cell Biol..

[3] Herrmann D (2014). Three-dimensional cancer models mimic cell-matrix interactions in the tumour microenvironment. Carcinogenesis.

[4] Bravo-Cordero JJ, Hodgson L, Condeelis J (2012). Directed cell invasion and migration during metastasis. Curr. Opin. Cell Biol..

[5] Atzori MG (2017). The anti-vascular endothelial growth factor receptor-1 monoclonal antibody D16F7 inhibits invasiveness of human glioblastoma and glioblastoma stem cells. J. Exp. Clin. Cancer Res..

[6] Gayan S, Teli A, Dey T (2017). Inherent aggressive character of invasive and non-invasive cells dictates the in vitro migration pattern of multicellular spheroid. Sci. Rep..

[7] Lim GJ, Kang SJ, Lee JY (2020). Novel invasion indices quantify the feed-forward facilitation of tumor invasion by macrophages. Sci. Rep..

[8] Cavaco ACM (2018). The interaction between laminin-332 and alpha3beta1 integrin determines differentiation and maintenance of CAFs, and supports invasion of pancreatic duct adenocarcinoma cells. Cancers (Basel).

[9] Celli JP (2014). An imaging-based platform for high-content, quantitative evaluation of therapeutic response in 3D tumour models. Sci. Rep..

[10] Kam Y, Guess C, Estrada L, Weidow B, Quaranta V (2008). A novel circular invasion assay mimics in vivo invasive behavior of cancer cell lines and distinguishes single-cell motility in vitro. BMC Cancer.

[11] Kleinman HK (1986). Basement membrane complexes with biological activity. Biochemistry.

[12] Hooper S, Marshall JF, Sahai E (2006). Tumor cell migration in three dimensions. Methods Enzymol..

[13] Zaman MH (2006). Migration of tumor cells in 3D matrices is governed by matrix stiffness along with cell-matrix adhesion and proteolysis. Proc. Natl. Acad. Sci. U.S.A..

[14] Poincloux R (2011). Contractility of the cell rear drives invasion of breast tumor cells in 3D Matrigel. Proc. Natl. Acad. Sci. U.S.A..

[15] Yu X, Machesky LM (2012). Cells assemble invadopodia-like structures and invade into matrigel in a matrix metalloprotease dependent manner in the circular invasion assay. PLoS ONE.

[16] Pijuan J (2019). In vitro cell migration, invasion, and adhesion assays: from cell imaging to data analysis. Front. Cell Dev. Biol..

[17] Hall DM, Brooks SA (2014). In vitro invasion assay using matrigel: a reconstituted basement membrane preparation. Methods Mol. Biol..

[18] Anguiano M (2017). Characterization of three-dimensional cancer cell migration in mixed collagen-Matrigel scaffolds using microfluidics and image analysis. PLoS ONE.

[19] Hulkower KI, Herber RL (2011). Cell migration and invasion assays as tools for drug discovery. Pharmaceutics.

[20] Kemper B, von Bally G (2008). Digital holographic microscopy for live cell applications and technical inspection. Appl. Opt..

[21] Marquet P (2005). Digital holographic microscopy: a noninvasive contrast imaging technique allowing quantitative visualization of living cells with subwavelength axial accuracy. Opt. Lett..

[22] Park Y, Depeursinge C, Popescu G (2018). Quantitative phase imaging in biomedicine. Nat. Photonics.

[23] Butkevich AN (2017). Hydroxylated fluorescent dyes for live-cell labeling: synthesis, spectra and super-resolution STED. Chemistry.

[24] El-Schich Z, Mölder AL, Wingren AG (2018). Quantitative phase imaging for label-free analysis of cancer cells—focus on digital holographic microscopy. Appl. Sci..

[25] Rappaz B, Breton B, Shaffer E, Turcatti G (2014). Digital holographic microscopy: a quantitative label-free microscopy technique for phenotypic screening. Comb. Chem. High Throughput Screen.

[26] Kim MK (2010). Principles and techniques of digital holographic microscopy. SPIE Rev..

[27] Rappaz B (2009). Noninvasive characterization of the fission yeast cell cycle by monitoring dry mass with digital holographic microscopy. J. Biomed. Opt..

[28] Toth AE (2014). Edaravone protects against methylglyoxal-induced barrier damage in human brain endothelial cells. PLoS ONE.

[29] Beigl TB, Hellesvik M, Saraste J, Arnesen T, Aksnes H (2020). N-terminal acetylation of actin by NAA80 is essential for structural integrity of the Golgi apparatus. Exp. Cell Res..

[30] Khmaladze A (2012). Cell volume changes during apoptosis monitored in real time using digital holographic microscopy. J. Struct. Biol..

[31] Mölder AL, Persson J, El-Schich Z, Czanner S, Gjorloff-Wingren A (2017). Supervised classification of etoposide-treated in vitro adherent cells based on noninvasive imaging morphology. J. Med. Imaging (Bellingham).

[32] Alm, K. *et al.* in *Holography—Basic Principles and Contemporary Applications* (ed Emilia Mihaylova) (IntechOpen, 2013).

[33] Kuhn J (2013). Label-free cytotoxicity screening assay by digital holographic microscopy. Assay Drug Dev. Technol..

[34] Lee K (2013). Quantitative phase imaging techniques for the study of cell pathophysiology: from principles to applications. Sensors (Basel).

[35] Marquet P, Depeursinge C, Magistretti PJ (2014). Review of quantitative phase-digital holographic microscopy: promising novel imaging technique to resolve neuronal network activity and identify cellular biomarkers of psychiatric disorders. Neurophotonics.

[36] Bettenworth D (2014). Quantitative stain-free and continuous multimodal monitoring of wound healing in vitro with digital holographic microscopy. PLoS ONE.

[37] Aksnes H, Marie M, Arnesen T, Drazic A (2018). Actin polymerization and cell motility are affected by NAA80-mediated posttranslational N-terminal acetylation of actin. Commun. Integr. Biol..

[38] Ju J (2017). NatD promotes lung cancer progression by preventing histone H4 serine phosphorylation to activate Slug expression. Nat. Commun..

[39] Lustig M, Zadka Y, Levitsky I, Gefen A, Benayahu D (2019). Adipocytes migration is altered through differentiation. Microsc. Microanal..

[40] Kemper B (2010). Label-free quantitative cell division monitoring of endothelial cells by digital holographic microscopy. J. Biomed. Opt..

[41] Zhang Y, Judson RL (2018). Evaluation of holographic imaging cytometer holomonitor M4(R) motility applications. Cytometry Part A J. Int. Soc. Anal. Cytol..

[42] Lenz P (2016). Multimodal quantitative phase imaging with digital holographic microscopy accurately assesses intestinal inflammation and epithelial wound healing. J. Vis. Exp..

[43] Rezaei M (2018). The expression of VE-cadherin in breast cancer cells modulates cell dynamics as a function of tumor differentiation and promotes tumor-endothelial cell interactions. Histochem. Cell Biol..

[44] Bettenworth D (2018). Quantitative phase microscopy for evaluation of intestinal inflammation and wound healing utilizing label-free biophysical markers. Histol. Histopathol..

[45] Langehanenberg P (2009). Automated three-dimensional tracking of living cells by digital holographic microscopy. J. Biomed. Opt..

[46] Dubois F (2006). Digital holographic microscopy for the three-dimensional dynamic analysis of in vitro cancer cell migration. J. Biomed. Opt..

[47] Tolde O (2018). Quantitative phase imaging unravels new insight into dynamics of mesenchymal and amoeboid cancer cell invasion. Sci. Rep..

[48] Simon B, Debailleul M, Beghin A, Tourneur Y, Haeberle O (2010). High-resolution tomographic diffractive microscopy of biological samples. J. Biophotonics.

[49] Gao Y (2017). Loss of ERalpha induces amoeboid-like migration of breast cancer cells by downregulating vinculin. Nat. Commun..

[50] Guo P, Huang J, Moses MA (2018). Quantitative phase imaging characterization of tumor-associated blood vessel formation on a chip. Proc. SPIE.

[51] Janicke B, Karsnas A, Egelberg P, Alm K (2017). Label-free high temporal resolution assessment of cell proliferation using digital holographic microscopy. Cytometry Part A J. Int. Soc. Anal. Cytol..

[52] Kamlund S, Strand D, Janicke B, Alm K, Oredsson S (2017). Influence of salinomycin treatment on division and movement of individual cancer cells cultured in normoxia or hypoxia evaluated with time-lapse digital holographic microscopy. Cell Cycle.

[53] Fallica B, Maffei JS, Villa S, Makin G, Zaman M (2012). Alteration of cellular behavior and response to PI3K pathway inhibition by culture in 3D collagen gels. PLoS ONE.

[54] Friedl P, Wolf K (2010). Plasticity of cell migration: a multiscale tuning model. J. Cell Biol..

[55] Loiselle JJ, Roy JG, Sutherland LC (2016). RBM5 reduces small cell lung cancer growth, increases cisplatin sensitivity and regulates key transformation-associated pathways. Heliyon.

[56] El-Schich Z, Nilsson E, Gerdtsson AS, Wingren C, Wingren AG (2015). Interfacing antibody-based microarrays and digital holography enables label-free detection for loss of cell volume. Future Sci. OA.

[57] Fridman R (1990). Reconstituted basement membrane (matrigel) and laminin can enhance the tumorigenicity and the drug resistance of small cell lung cancer cell lines. Proc. Natl. Acad. Sci. U.S.A..

[58] Li Y, Petrovic L, La J, Celli JP, Yelleswarapu CS (2014). Digital holographic microscopy for longitudinal volumetric imaging of growth and treatment response in three-dimensional tumor models. J. Biomed. Opt..

[59] Charrière F (2007). Sub-cellular quantitative optical diffraction tomography with digital holographic microscopy. Proc. SPIE.

[60] Muschol M, Wenders C, Wennemuth G (2018). Four-dimensional analysis by high-speed holographic imaging reveals a chiral memory of sperm flagella. PLoS ONE.

[61] Sebesta M (2016). HoloMonitor M4: holographic imaging cytometer for real-time kinetic label-free live-cell analysis of adherent cells. SPIE Proc..

[62] Phase Holographic Imaging, P. H. I. A. B. HoloMonitor® M4 Setup and Operation Manual. https://phiab.com/reports/manuals/M4-SetupAndOperationManual.pdf (2018).

[63] Wiame E (2018). NAT6 acetylates the N-terminus of different forms of actin. FEBS J..

