# Author Correction: Exploiting the potential of commercial digital holographic microscopy by combining it with 3D matrix cell culture assays

**DOI:** 10.1038/s41598-023-27960-2

**Published:** 2023-01-20

**Authors:** Monica Hellesvik, Hanne Øye, Henriette Aksnes

**Affiliations:** 1 Department of Biomedicine, University of Bergen, Bergen, Norway; 2 Department of Biological Sciences, University of Bergen, Bergen, Norway

Correction to: *Scientific Reports* 10.1038/s41598-020-71538-1, published online 07 September 2020

The original version of this Article contained errors in the legends of Figure 1 and 2, where 1% Matrigel should read 50% Matrigel.

Figure 1. “Holographic imaging of Matrigel embedded cells reveal the impact of culture extracellular environment on cell morphology and cell proliferation. (a) U2OS cells were prepared for live-cell holographic imaging and seeded on uncoated surface, seeded in 1% Matrigel or embedded in 50% Matrigel followed by image acquisition each 15 min at 20 × for 16 h using the DHM system. The cyan-yellow-white colour bar in the pseudo coloured holographic 2D representation of the 3D images displays a vertical scale (z-plane) correlating the image colouring with optical thickness. (b) Zoom-in 3D view revealing morphological differences in U2OS cells as effect of Matrigel in the extracellular environment. (c–e) Quantification of the morphological features cell spread area, cell shape irregularity and cell optical volume in U2OS cells seeded as in (a). Three randomly chosen fields of view from each of three wells per condition were analyzed. Images at timepoint 17.5 h post Matrigel embedding of cells was further processed and analyzed using the integrated software. Data are plotted as lognormal for improved visualization. Original units for the cell morphological parameters are displayed on the y-axis by means of logarithmic scale. Shown is the mean ± SEM of three independent experiments pooled together. Mean of Cell spreading area = 847.7 um2 (Uncoated); 1150 um2 (1% Matrigel); and mean = 809.3 um2 (1% Matrigel). Mean of Cell shape irregularity = 0.4326 (Uncoated); 0.5454 (1% Matrigel); and mean = 0.4339 (1% Matrigel). Mean of Cell optical volume = 2,246um3 (Uncoated); 2130 um3 (1% Matrigel); and mean = 2321 um3 (1% Matrigel). n = 897 (Uncoated); n = 1,170 (1% Matrigel); and n = 891 (50% Matrigel). ns not significant, *p ≤ 0.05; **p ≤ 0.01; ****p ≤ 0.0001, one-way ANOVA with Dunnett’s multiple comparison test. (f) Quantification of cell proliferation comparing relative increase in cell numbers between t0 = first image and last image 16 h later acquired as in (a). For each condition; single cells in four randomly chosen fields of view from three replicate wells in each experiment were counted from the holographic images using the integrated software. Shown is the mean ± SEM of three independent experiments pooled together. Mean of Cell proliferation = 168% (Uncoated); 170.2% (1% Matrigel); and mean = 157.5% (1% Matrigel). n = 36 (Uncoated); n = 36 (1% Matrigel); and n = 36 (50% Matrigel). ns not significant, *p ≤ 0.05, one-way ANOVA with Dunnett’s multiple comparison test.”

now reads:

Figure 1. “Holographic imaging of Matrigel embedded cells reveal the impact of culture extracellular environment on cell morphology and cell proliferation. (**a**) U2OS cells were prepared for live-cell holographic imaging and seeded on uncoated surface, seeded in 1% Matrigel or embedded in 50% Matrigel followed by image acquisition each 15 min at 20 × for 16 h using the DHM system. The cyan-yellow-white colour bar in the pseudo coloured holographic 2D representation of the 3D images displays a vertical scale (z-plane) correlating the image colouring with optical thickness. (**b**) Zoom-in 3D view revealing morphological differences in U2OS cells as effect of Matrigel in the extracellular environment. (**c**–**e**) Quantification of the morphological features cell spread area, cell shape irregularity and cell optical volume in U2OS cells seeded as in (**a**). Three randomly chosen fields of view from each of three wells per condition were analyzed. Images at timepoint 17.5 h post Matrigel embedding of cells was further processed and analyzed using the integrated software. Data are plotted as lognormal for improved visualization. Original units for the cell morphological parameters are displayed on the y-axis by means of logarithmic scale. Shown is the mean ± SEM of three independent experiments pooled together. Mean of Cell spreading area = 847.7 um^2^ (Uncoated); 1150 um^2^ (1% Matrigel); and mean = 809.3 um^2^ (50% Matrigel). Mean of Cell shape irregularity = 0.4326 (Uncoated); 0.5454 (1% Matrigel); and mean = 0.4339 (50% Matrigel). Mean of Cell optical volume = 2246 um^3^ (Uncoated); 2130 um^3^ (1% Matrigel); and mean = 2321 um^3^ (50% Matrigel). n = 897 (Uncoated); n = 1170 (1% Matrigel); and n = 891 (50% Matrigel). *ns* not significant, *p ≤ 0.05; **p ≤ 0.01; ****p ≤ 0.0001, one-way ANOVA with Dunnett’s multiple comparison test. (**f**) Quantification of cell proliferation comparing relative increase in cell numbers between t_0_ = first image and last image 16 h later acquired as in (**a**). For each condition; single cells in four randomly chosen fields of view from three replicate wells in each experiment were counted from the holographic images using the integrated software. Shown is the mean ± SEM of three independent experiments pooled together. Mean of Cell proliferation = 168% (Uncoated); 170.2% (1% Matrigel); and mean = 157.5% (50% Matrigel). n = 36 (Uncoated); n = 36 (1% Matrigel); and n = 36 (50% Matrigel). *ns* not significant, *p ≤ 0.05, one-way ANOVA with Dunnett’s multiple comparison test.”

Figure 2. “Holographic imaging of cells reveal the impact of culture extracellular environment on cell migration and cell motility. U2OS single cell tracking analysis performed with the integrated software from the phase shift holographic images acquired as described in Fig. 1. Cells were prepared for live-cell holographic imaging and seeded on uncoated surface, seeded in 1% Matrigel or embedded in 50% Matrigel followed by image acquisition every 15 min at 20 × for in total 16 h using the DHM system. (**a**–**c**) Single cell 2D movement trajectories from 10 to 13 cells per field of view were displayed as a rose plot. Each plot represents migration path over 16 h. (**d**–**f**) Cell motility speed, cell motility and cell migration were quantified from single cell tracking data. Motility distance was defined as the accumulated movement of the cell over time from the starting point to the end point of the cell path. Migration was defined as the shortest distance from the starting point and the end point of the cell path. Each data point represents the quantitative data from tracking a single cell throughout 16 h of imaging. Between 10 and 13 cells from one randomly chosen field of view from two wells per condition were analyzed using the integrated software. Data are plotted as lognormal for improved visualization. Original units for cell movement parameters are displayed on the y-axis by means of logarithmic scale. Shown is the mean ± SEM of three independent experiments pooled together. Mean of Cell motility speed = 9.115 µm/h (Uncoated); 22.57 µm/h (1% Matrigel); and mean = 9.328 µm/h (1% Matrigel). Mean of Cell motility = 143.3 µm (Uncoated); 356.8 µm (1% Matrigel); and mean = 145 µm (1% Matrigel). Mean of Cell migration = 20.3 µm (Uncoated); 54.40 µm (1% Matrigel); and mean = 12.13 µm (1% Matrigel). n = 69 (Uncoated); n = 72 (1% Matrigel); and n = 68 (50% Matrigel). *ns* not significant, *p ≤ 0.05; ****p ≤ 0.0001, one-way ANOVA with Dunnett’s multiple comparison test.”

now reads:

Figure 2. “Holographic imaging of cells reveal the impact of culture extracellular environment on cell migration and cell motility. U2OS single cell tracking analysis performed with the integrated software from the phase shift holographic images acquired as described in Fig. 1. Cells were prepared for live-cell holographic imaging and seeded on uncoated surface, seeded in 1% Matrigel or embedded in 50% Matrigel followed by image acquisition every 15 min at 20 × for in total 16 h using the DHM system. (**a**–**c**) Single cell 2D movement trajectories from 10 to 13 cells per field of view were displayed as a rose plot. Each plot represents migration path over 16 h. (**d**–**f**) Cell motility speed, cell motility and cell migration were quantified from single cell tracking data. Motility distance was defined as the accumulated movement of the cell over time from the starting point to the end point of the cell path. Migration was defined as the shortest distance from the starting point and the end point of the cell path. Each data point represents the quantitative data from tracking a single cell throughout 16 h of imaging. Between 10 and 13 cells from one randomly chosen field of view from two wells per condition were analyzed using the integrated software. Data are plotted as lognormal for improved visualization. Original units for cell movement parameters are displayed on the y-axis by means of logarithmic scale. Shown is the mean ± SEM of three independent experiments pooled together. Mean of Cell motility speed = 9.115 µm/h (Uncoated); 22.57 µm/h (1% Matrigel); and mean = 9.328 µm/h (50% Matrigel). Mean of Cell motility = 143.3 µm (Uncoated); 356.8 µm (1% Matrigel); and mean = 145 µm (50% Matrigel). Mean of Cell migration = 20.3 µm (Uncoated); 54.40 µm (1% Matrigel); and mean = 12.13 µm (50% Matrigel). n = 69 (Uncoated); n = 72 (1% Matrigel); and n = 68 (50% Matrigel). *ns* not significant, *p ≤ 0.05; ****p ≤ 0.0001, one-way ANOVA with Dunnett’s multiple comparison test.”

The original Article has been corrected.

