# Supplementary material for: Exploiting the potential of commercial digital holographic microscopy by combining it with 3D matrix cell culture assays
Source: Sci Rep. 2020 Sep 7;10:14680. doi: 10.1038/s41598-020-71538-1 (PMC7477226; doi:10.1038/s41598-020-71538-1)
Supplement: Supplementary file 1 — Supplementary Legends. [file 41598_2020_71538_MOESM1_ESM.pdf]

SUPPLEMENTARY INFORMATION

Exploiting the potential of commercial digital holographic microscopy by combining it with 3D matrix cell culture assays

Monica Hellesvik[1,2], Hanne Øye[1], Henriette Aksnes[1]*

Overview supplementary information:

Supplementary Figure S1 (accompanying Figure 1)
Workflow for cell identification for morphological parameters analysis.

Supplementary Figure S2 (accompanying Figure 2)
Holographic imaging reveal the impact of culture extracellular environment on cell motility trajectories.

Supplementary Movies S1-3 (accompanying Figure 1)

Movie S1. U2OS Uncoated

Movie S2. U2OS 1 % Matrigel

Movie S3. U2OS 50 % Matrigel

Supplementary Movies S4-7 (accompanying Figure 4)

Movie S4. U2OS wound healing migration 2D view

Movie S5. U2OS wound healing migration 3D view

Movie S6. U2OS wound healing invasion assay 2D view

Movie S7. U2OS wound healing invasion assay 3D view
