# Supplementary figures and images for: Exploiting the potential of commercial digital holographic microscopy by combining it with 3D matrix cell culture assays
Source: Sci Rep. 2020 Sep 7;10:14680. doi: 10.1038/s41598-020-71538-1 (PMC7477226; doi:10.1038/s41598-020-71538-1)

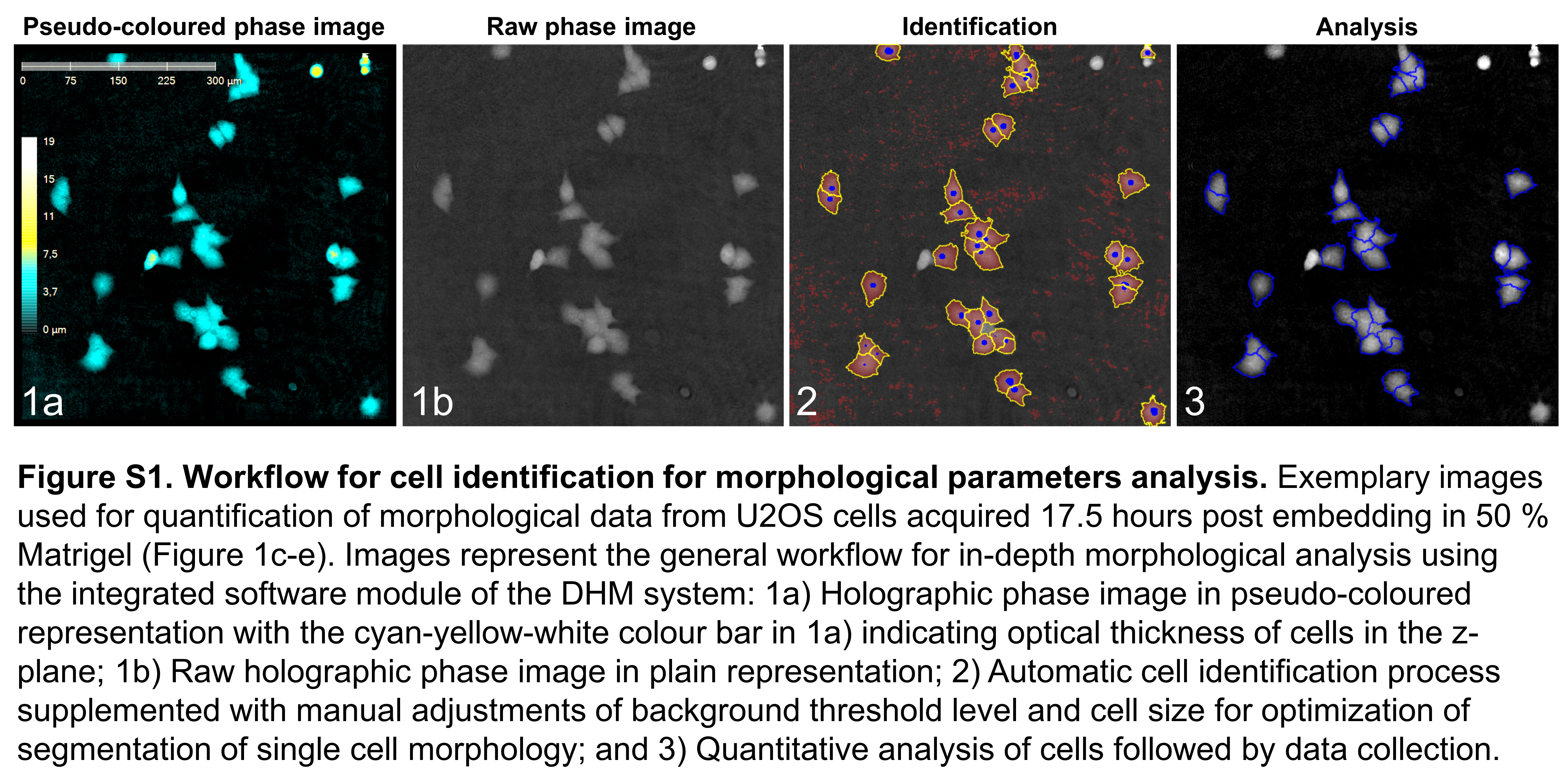

Supplement: Supplementary file 2 — Supplementary Figure S1. [file 41598_2020_71538_MOESM2_ESM.tif]

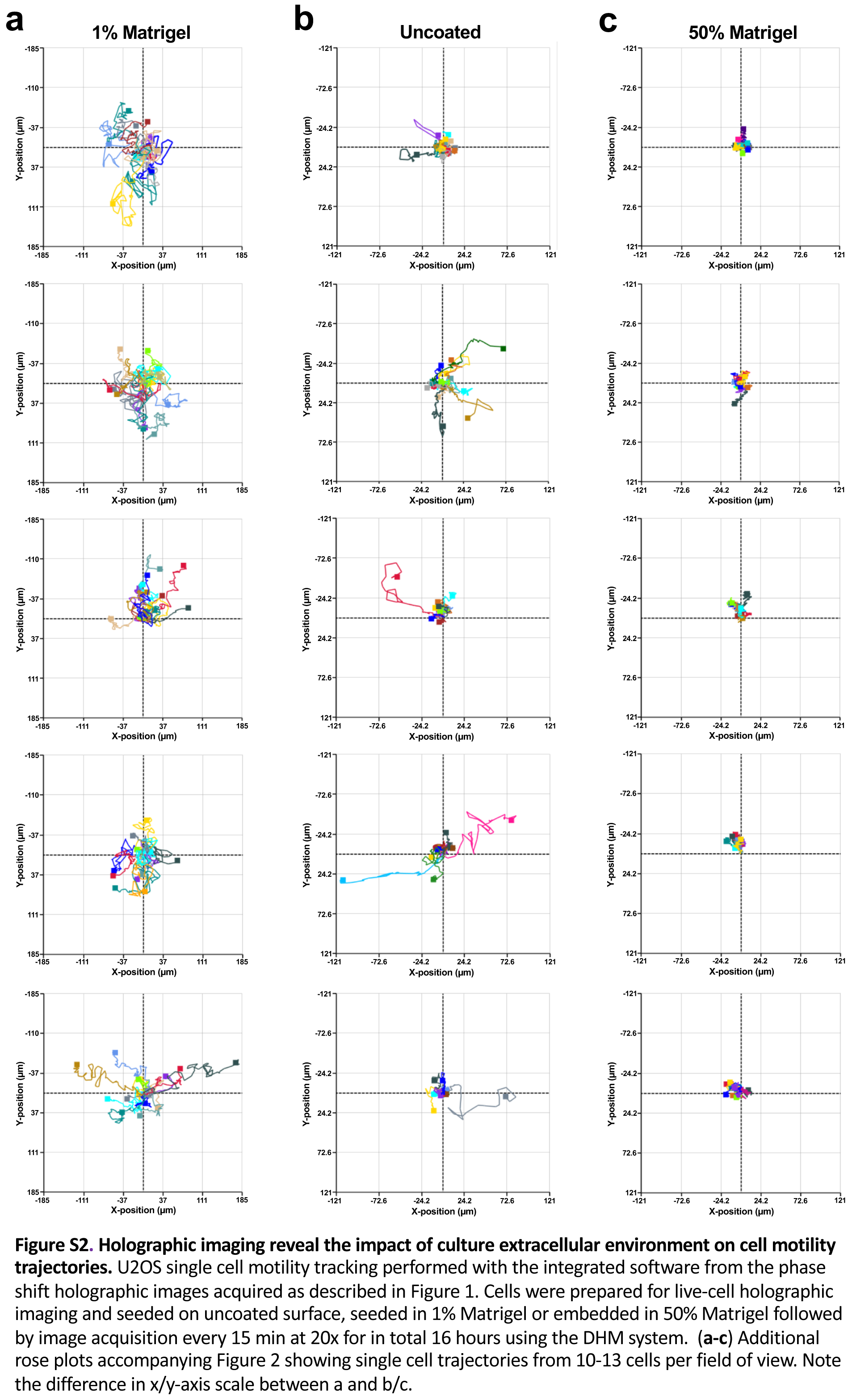

Supplement: Supplementary file 3 — Supplementary Figure S2. [file 41598_2020_71538_MOESM3_ESM.tif]
